# Association between the C-reactive protein–triglyceride–glucose index and coronary heart disease in metabolic dysfunction-associated steatotic liver disease patients: a cross-sectional study

**DOI:** 10.3389/fendo.2026.1874886

**Published:** 2026-07-07

**Authors:** Kaiyue Shen, Jiajia Zhao, Kunkun Li, Lidong Xu, Huili Wu

**Affiliations:** Department of Gastroenterology, Zhengzhou Central Hospital Affiliated to Zhengzhou University, Zhengzhou, Henan, China

**Keywords:** coronary heart disease, C-reactive protein–triglyceride–glucose index, insulin resistance, metabolic dysfunction-associated steatotic liver disease, nomogram

## Abstract

**Background:**

Metabolic dysfunction-associated steatotic liver disease (MASLD) is strongly associated with coronary heart disease (CHD), but convenient and effective biomarkers for identifying CHD risk in patients with MASLD remain limited. The C-reactive protein–triglyceride–glucose index (CTI) reflects both systemic inflammation and insulin resistance, and may add value to cardiovascular risk stratification. This study examined the association of CTI with the presence and severity of CHD in patients with MASLD and developed a nomogram for individualized CHD assessment.

**Methods:**

This study included 611 patients with MASLD who underwent coronary angiography. Participants were classified according to coronary angiography findings. Logistic regression was first performed to assess the association between CTI and CHD, followed by subgroup and restricted cubic spline (RCS) analyses. Among patients with CHD, the association between CTI and coronary lesion severity was assessed. Receiver operating characteristic (ROC) analysis and DeLong’s test were applied to compare the discriminative performance of CTI with other simple insulin resistance-related indices. Independent predictors of CHD were identified using the least absolute shrinkage and selection operator (LASSO) regression and multivariable logistic regression, and a nomogram model was subsequently established and internally validated.

**Results:**

Among the 611 patients with MASLD, 387 (63.3%) had CHD. CTI was independently associated with odds of CHD (OR 2.48, 95% CI 1.69–3.63, P < 0.001) and higher CTI levels corresponded to greater coronary stenosis severity. The association between CTI and CHD was generally stable across common subgroups, but it was stronger in patients aged ≥55 years. CTI showed significantly better discriminative ability for CHD than other simple indices of insulin resistance. Smoking status, age, neutrophils (NEUT), non-high-density lipoprotein cholesterol (non-HDL-C), serum albumin (ALB), CTI, and subclinical carotid atherosclerosis (SCAS) were included in the final nomogram. The AUC was 0.845 (95% CI 0.815–0.876), indicating good discrimination for CHD.

**Conclusions:**

Higher CTI was associated with CHD presence and greater coronary lesion burden among MASLD patients. The nomogram may help identify patients who warrant further cardiovascular evaluation.

## 1 Introduction

Coronary heart disease (CHD) is primarily driven by atherosclerotic stenosis of the coronary arteries. As plaques progress or become unstable, coronary blood flow may be reduced or interrupted, potentially leading to myocardial infarction (1). In China, approximately 11.39 million people are affected by CHD. As the number of cases continues to rise, CHD has become an important public health issue and places a heavy economic burden on both patients and society (2).

Metabolic dysfunction-associated steatotic liver disease (MASLD) is a chronic liver disorder that occurs in the context of systemic metabolic abnormalities, and was formerly referred to as non-alcoholic fatty liver disease (NAFLD) or metabolic dysfunction-associated fatty liver disease (MAFLD) (3). The updated terminology places metabolic dysfunction at the core of MASLD, with obesity, diabetes mellitus, hypertension, and dyslipidemia regarded as key related conditions. These metabolic disturbances not only underlie the development of MASLD but also represent major determinants of cardiovascular risk (4). MASLD now affects more than 30% of adults worldwide, and its increasing prevalence has made it an important global health concern (5).

MASLD is increasingly recognized as a disease closely connected with cardiometabolic disorders (6). Compelling evidence supports a substantial association between MASLD and increased CHD morbidity and mortality (7, 8). MASLD and CHD share several pathophysiological mechanisms, including insulin resistance (IR), systemic inflammation, altered lipoprotein metabolism, endothelial dysfunction, and increased thrombogenicity (9). Patients with MASLD often exhibit marked disturbances in glucose and lipid metabolism (10). Increased intrahepatic lipid accumulation can further aggravate IR (11). In turn, IR may impair vascular endothelial function and accelerate thrombus formation by activating inflammatory pathways, elevating oxidative stress, and promoting platelet adhesion and aggregation (12, 13). Chronic low-grade inflammation is another important feature of MASLD (14). Harmful lipid accumulation can activate intrahepatic immune responses and upregulate pro-inflammatory cytokines. This inflammatory response can induce the hepatic acute-phase reaction and increase the synthesis of acute-phase proteins, including C-reactive protein (CRP) and fibrinogen (15). These inflammatory mediators may facilitate macrophage uptake of lipoproteins, thereby promoting foam cell formation and contributing to atherosclerotic plaque formation and progression (16, 17).

As a classical indicator of systemic inflammation, CRP is strongly correlated with both the initiation and progression of coronary atherosclerosis (18, 19). Similarly, the triglyceride-glucose (TyG) index is widely used as a simple surrogate for insulin resistance (20). Previous studies have reported associations between the TyG index and atherosclerosis, CHD risk, coronary lesion severity, and all-cause mortality (21). Besides TyG, other simple insulin resistance-related indices, including METS-IR (the metabolic score for insulin resistance) (22), TG/HDL-C (the triglyceride/high-density lipoprotein cholesterol ratio) (23), and TyG-BMI (triglyceride glucose-body mass index) (24), have also been widely used for cardiometabolic risk stratification.

Single metabolic or inflammatory markers may not fully capture cardiovascular risk. Therefore, composite indices that incorporate both metabolic and inflammatory features have attracted increasing attention in cardiovascular risk assessment (25, 26). CTI was first described by Ruan et al. in 2022 as an index derived from CRP and the TyG index, thereby linking systemic inflammation with insulin resistance in a single measure (27). Subsequent studies have demonstrated that CTI performs well in predicting hypertension, stroke, incident cardiovascular disease, and adverse outcomes (27–29).

Despite the growing interest in CTI, its role in CHD assessment among patients with MASLD remains understudied. Thus, the present study was designed to investigate the association between CTI and CHD in this population. We further aimed to evaluate whether CTI could provide additional information for cardiovascular risk assessment in these high-risk individuals.

## 2 Materials and methods

### 2.1 Study design and participants

In this retrospective cross-sectional study, 611 individuals with MASLD at admission were included. All participants had complete clinical and laboratory data and underwent coronary angiography at Zhengzhou Central Hospital Affiliated to Zhengzhou University between January 2024 and December 2025. According to coronary angiographic findings, the participants were subsequently stratified into the MASLD group and the MASLD+CHD group. The exclusion criteria were: (1) viral hepatitis, drug-induced liver injury, autoimmune hepatitis, or other known liver diseases; (2) regular statin use for ≥3 months before admission; (3) acute infection, such as respiratory or urinary tract infection, or chronic inflammatory conditions, including rheumatoid arthritis and inflammatory bowel disease; (4) history of congenital heart disease, prior coronary revascularization, previous cardiac procedures, or other cardiac diseases; and (5) hematologic disorders, autoimmune diseases, malignant tumors, or a history of severe cerebrovascular disease.

The Ethics Committee of Zhengzhou Central Hospital Affiliated to Zhengzhou University approved this study (Approval No. ZXYY2026059). The study was conducted in accordance with the Declaration of Helsinki. Because of the retrospective design, written informed consent was waived.

### 2.2 Definitions

According to the latest EASL-EASD-EASO guidelines, MASLD was diagnosed based on hepatic steatosis detected by qualified and experienced ultrasonographers using standardized ultrasound criteria, after excluding other causes of steatosis and excessive alcohol intake per week (≥140–350 g/wk in women and ≥210–420 g/wk in men). Additionally, patients must have had at least one of five cardiometabolic risk factors: (1) BMI ≥ 23 kg/m² or waist circumference ≥ 90 cm (male)/≥ 80 cm (female); (2) fasting glucose ≥ 100.80 mg/dL (5.6 mmol/L), or a history of diabetes mellitus or glucose-lowering treatment; (3) SBP ≥ 130 mmHg and/or DBP ≥ 85 mmHg, a hypertension diagnosis, or antihypertensive medication use; (4) TG levels ≥ 150 mg/dL (1.70 mmol/L), or receipt of lipid-modifying therapy; and (5) HDL-C < 38.67 mg/dL (1.0 mmol/L) in men and < 50.27 mg/dL (1.3 mmol/L) in women, or use of lipid-lowering agents(6).

CHD was diagnosed when coronary angiography showed ≥50% stenosis in one or more major epicardial coronary arteries or their main branches. Coronary angiography (CAG) was performed by more than one experienced interventional cardiologist using the Judkins technique (30). The angiographic findings were independently reviewed by at least two experienced cardiologists. The diseased-vessel number was determined by counting major coronary arteries with ≥50% stenosis and was classified as 1-, 2-, or 3-vessel disease. The Gensini score was used to quantify coronary lesion burden. Weighted points were assigned based on stenosis grade and lesion site, with a greater total score indicating more advanced coronary artery disease (31).

Subclinical carotid atherosclerosis (SCAS) was diagnosed based on cIMT > 1.0 mm and/or the presence of carotid plaque (32). Carotid ultrasonography was performed using a 12–5 MHz probe to examine the common, internal, and external carotid arteries and the bifurcation. The mean cIMT value was obtained from the far wall of the common carotid artery, close to the carotid bulb. Carotid plaque was identified when a focal lesion extended ≥0.5 mm into the arterial lumen, exceeded 50% of the adjacent IMT, or had an IMT measurement >1.5 mm (33). All scans were conducted by experienced sonographers according to the recommended standardized protocol (34).

### 2.3 Data collection

Baseline data were retrospectively extracted from the Hospital Information System during hospitalization, including demographic characteristics (age and gender), clinical history [smoking status, hypertension (HT), diabetes mellitus (DM)], and anthropometric measurements (height and weight). Laboratory data were obtained from the first fasting venous blood sample after admission, including CRP, neutrophil count (NEUT), platelet count (PLT), red blood cell count (RBC), hemoglobin (Hb), hematocrit (Hct), glycated hemoglobin (HbA1c), fasting plasma glucose (FPG), serum albumin (ALB), alanine aminotransferase (ALT), aspartate aminotransferase (AST), total cholesterol (TC), TG, HDL-C, low-density lipoprotein cholesterol (LDL-C), non-high-density lipoprotein cholesterol (non-HDL-C), blood urea nitrogen (BUN), uric acid (UA), estimated glomerular filtration rate (eGFR), homocysteine (Hcy), and thyroid-stimulating hormone (TSH).

The indices were calculated using the following formulas:


$$\text{BMI }=\text{ weight }(\text{kg})\;/{\text{ height}}^{2}\;({\text{m}}^{2})$$

TyG=ln [TG[mg/dL]×FPG[mg/dL]/2]

CTI=0.412×ln[CRP (mg/L)]+TyG

TyG − BMI= TyG × BMI

$$\text{METS}-\text{IR}=\text{ln}[(2\times \text{FPG}[\text{mg}/\text{dL}])+\text{TG}[\text{mg}/\text{dL}]]\times \text{BMI}[\text{kg}/{\text{m}}^{2}]/\text{ln}[\text{HDL}-\text{C}(\text{mg}/\text{dL})]$$

TG/HDL−C=TG[mg/dL]/HDL−C[mg/dL]

### 2.4 Statistical analysis

Continuous variables were tested for normality with the Shapiro–Wilk test. Normally distributed data were shown as mean ± SD and compared by independent-samples t test; non-normally distributed data were shown as median (IQR), and compared via the Mann–Whitney U test. Categorical data were presented as counts (percentages) and analyzed using the chi-square test.

Binary logistic regression was used to evaluate CTI in relation to CHD. CTI was analyzed both as a continuous variable and by quartiles, using the first quartile as the reference. Results were reported as odds ratios (ORs) with 95% confidence intervals (CIs). Three models were fitted: a crude model; a model adjusted for BMI, age, gender, smoking status, HT, and DM; and a fully adjusted model that additionally included NEUT, HbA1c, TC, HDL-C, ALB, ALT, AST, and UA. To examine potential nonlinearity in the association between CTI and CHD, restricted cubic spline (RCS) analysis was performed. Subgroup analyses were performed according to age (<55 vs. ≥55 years), gender, hypertension, diabetes mellitus, and smoking status. Spearman’s correlation and quantile regression were used to assess the relationship between CTI and the Gensini score among patients with CHD. Differences in CTI across categories of diseased-vessel number were assessed with the Kruskal–Wallis test. The discriminative ability of CTI and other simple insulin resistance-related indices to identify CHD in MASLD patients was evaluated through ROC curves, with AUC differences tested using DeLong’s method.

Candidate predictors were first screened using LASSO regression. The retained variables were then included in a multivariable logistic regression model to determine independent factors related to CHD. A nomogram was constructed based on the final predictors. Model performance was evaluated by the area under the curve (AUC), C-index, bootstrap internal validation, calibration curve, decision curve analysis (DCA), and clinical impact curve (CIC). The incremental value of CTI was assessed by comparing a base model without CTI and a full model with CTI, using integrated discrimination improvement (IDI) and continuous net reclassification improvement (NRI).

All statistical analyses were conducted using R 4.5.2.

## 3 Results

### 3.1 Baseline characteristics

**Table 1** shows the baseline characteristics of the study cohort. Of the 611 patients with MASLD, 387 were classified into the MASLD+CHD group, and 224 into the MASLD group. Compared with the MASLD group, patients in the MASLD+CHD group were more frequently male (67.44% vs. 58.04%, P = 0.025) and had a higher median age (58.00 [51.00, 65.00] vs. 54.00 [45.00, 61.00], P < 0.001). In addition, hypertension, diabetes mellitus, smoking, and SCAS were more common in the MASLD+CHD group (62.02% vs. 50.45%, P = 0.007; 40.31% vs. 15.62%, P < 0.001; 26.36% vs. 10.27%, P < 0.001; and 85.27% vs. 64.29%, P < 0.001, respectively). Several laboratory parameters, including CRP, NEUT, HbA1c, FPG, TC, TG, non-HDL-C, BUN, UA, and Hcy, were observed at higher levels in the MASLD+CHD group (all P < 0.05). By contrast, lower levels of HDL-C, ALB, eGFR, and TSH were observed in the MASLD+CHD group (all P < 0.05). Patients in the MASLD+CHD group had higher CTI values than those in the MASLD group (9.83 [9.37, 10.36] vs. 9.01 [8.57, 9.50], P < 0.001). Likewise, TyG, TG/HDL-C, METS-IR, and TyG-BMI were all significantly higher in the MASLD+CHD group than in the MASLD group (all P < 0.001).

**Table 1:** Baseline characteristics of the study participants.

Variable	Overall	MASLD	MASLD+CHD	P value
n	611	224	387	
Male, %	391 (63.99)	130 (58.04)	261 (67.44)	0.025
Age, years	56.00 [48.00, 63.00]	54.00 [45.00, 61.00]	58.00 [51.00, 65.00]	< 0.001
BMI, kg/m²	27.01 [24.77, 29.00]	26.98 [24.74, 28.87]	27.01 [24.99, 29.05]	0.943
Smoking, %	125 (20.46)	23 (10.27)	102 (26.36)	< 0.001
Hypertension, %	353 (57.77)	113 (50.45)	240 (62.02)	0.007
Diabetes mellitus, %	191 (31.26)	35 (15.62)	156 (40.31)	< 0.001
SCAS, %	474 (77.58)	144 (64.29)	330 (85.27)	< 0.001
CRP, mg/L	1.87 [1.08, 3.67]	1.33 [0.40, 2.31]	2.54 [1.38, 4.65]	< 0.001
NEUT, ×10^9/L	4.11 [3.25, 5.33]	3.63 [2.98, 4.50]	4.39 [3.43, 5.94]	< 0.001
PLT, ×10^9/L	225.00 [190.00, 266.00]	225.00 [190.00, 269.00]	225.00 [189.50, 264.50]	0.866
RBC, ×10^12/L	4.75 (0.52)	4.70 (0.48)	4.78 (0.54)	0.088
Hb, g/L	145.00 [133.00, 155.00]	143.00 [133.00, 153.00]	145.00 [133.00, 156.00]	0.131
Hct, %	43.86 (4.68)	43.66 (4.35)	43.98 (4.86)	0.407
HbA1c, %	6.00 [5.60, 6.75]	5.80 [5.50, 6.12]	6.20 [5.70, 7.50]	< 0.001
FPG, mmol/L	6.04 [5.33, 7.55]	5.58 [5.10, 6.17]	6.53 [5.54, 8.99]	< 0.001
TC, mmol/L	4.90 [4.20, 5.56]	4.67 [3.97, 5.44]	4.98 [4.30, 5.67]	0.002
TG, mmol/L	2.01 [1.42, 2.89]	1.63 [1.20, 2.44]	2.21 [1.58, 3.09]	< 0.001
HDL-C, mmol/L	1.00 [0.86, 1.17]	1.09 [0.92, 1.27]	0.97 [0.83, 1.12]	< 0.001
LDL-C, mmol/L	2.67 [2.06, 3.24]	2.56 [2.01, 3.15]	2.72 [2.08, 3.30]	0.054
non-HDL-C, mmol/L	3.87 [3.16, 4.49]	3.61 [2.94, 4.31]	3.98 [3.30, 4.62]	< 0.001
ALB, g/L	42.90 [40.65, 45.10]	44.30 [41.80, 45.80]	42.40 [39.50, 44.50]	< 0.001
ALT, U/L	25.00 [18.00, 37.50]	26.00 [18.00, 38.00]	25.00 [17.00, 37.00]	0.805
AST, U/L	22.00 [18.00, 29.00]	21.50 [18.00, 26.00]	22.00 [17.00, 30.00]	0.246
BUN, mmol/L	5.44 [4.57, 6.54]	5.20 [4.39, 6.30]	5.59 [4.64, 6.66]	0.010
UA, μmol/L	360.50 [303.60, 421.60]	348.55 [286.67, 407.30]	369.80 [311.35, 431.95]	0.004
eGFR, mL/min/1.73 m²	100.71 [91.54, 108.87]	103.66 [96.35, 110.66]	98.07 [89.50, 108.18]	< 0.001
Hcy, μmol/L	12.00 [10.00, 16.00]	12.00 [9.45, 15.00]	12.10 [10.00, 17.00]	0.015
TSH, mIU/L	1.66 [1.18, 2.43]	1.90 [1.23, 2.58]	1.51 [1.09, 2.27]	0.009
FIB, g/L	2.89 [2.40, 3.45]	2.74 [2.33, 3.29]	2.97 [2.46, 3.57]	0.001
CTI	9.55 [9.00, 10.16]	9.01 [8.57, 9.50]	9.83 [9.37, 10.36]	< 0.001
TyG	9.26 [8.83, 9.67]	8.92 [8.55, 9.32]	9.43 [9.05, 9.84]	< 0.001
TG/HDL-C	4.62 [2.92, 7.26]	3.49 [2.27, 5.81]	5.21 [3.50, 7.93]	< 0.001
METS-IR	44.62 [40.33, 49.33]	42.36 [38.52, 47.48]	45.87 [41.44, 50.44]	< 0.001
TyG-BMI	248.75 [227.44, 271.23]	238.22 [220.54, 263.05]	255.00 [233.45, 276.12]	< 0.001

P < 0.05 was considered statistically significant.

Data are presented as mean (SD), median [IQR], or n (%). BMI, body mass index; SCAS, subclinical carotid atherosclerosis; CRP, C-reactive protein; NEUT, neutrophil count; PLT, platelet count; RBC, red blood cell count; Hb, hemoglobin; Hct, hematocrit; HbA1c, glycated hemoglobin; FPG, fasting plasma glucose; TC, total cholesterol; TG, triglycerides; HDL-C, high-density lipoprotein cholesterol; LDL-C, low-density lipoprotein cholesterol; non-HDL-C, non-high-density lipoprotein cholesterol; ALB, albumin; ALT, alanine aminotransferase; AST, aspartate aminotransferase; UA, uric acid; eGFR, estimated glomerular filtration rate; Hcy, homocysteine; TSH, thyroid-stimulating hormone; FIB, fibrinogen; CTI, C-reactive protein–triglyceride–glucose index; TyG, triglyceride–glucose index; METS-IR, metabolic score for insulin resistance; TyG-BMI: triglyceride glucose-body mass index.

### 3.2 Baseline characteristics according to CTI quartiles

To further examine the association between CTI and CHD, CTI was categorized into quartiles. Baseline characteristics stratified by CTI quartiles are presented in **Table 2**. Overall, higher CTI quartiles were associated with a less favorable inflammatory and metabolic profile. The proportions of patients with smoking, diabetes mellitus, SCAS, and CHD increased across CTI quartiles, with the proportion of CHD increasing markedly from 28.8% in Q1 to 87.5% in Q4. In addition, CRP, NEUT, HbA1c, FPG, TC, TG, non-HDL-C, UA, ALT, AST, and FIB levels were higher in the upper CTI quartiles, whereas HDL-C and ALB levels were lower (all P < 0.05).

**Table 2:** Baseline characteristics according to CTI quartiles.

Variable	CTI quartiles (n=611)				P value
	Q1 (lowest)	Q2	Q3	Q4 (highest)	
	(n=153)	(n=153)	(n=153)	(n=152)	
Male, %	89 (58.2%)	102 (66.7%)	96 (62.7%)	104 (68.4%)	0.248
Age, years	55.00 (48.00–61.00)	56.00 (49.00–63.00)	58.00 (50.00–64.00)	55.00 (44.00–64.00)	0.279
BMI, kg/m²	26.67 (24.49–28.10)	27.40 (25.04–29.93)	26.83 (25.00–28.40)	26.96 (24.77–29.06)	0.065
Smoking, %	18 (11.8%)	31 (20.3%)	37 (24.2%)	39 (25.7%)	0.012
Hypertension, %	82 (53.6%)	82 (53.6%)	90 (58.8%)	99 (65.1%)	0.131
Diabetes mellitus, %	15 (9.8%)	27 (17.6%)	62 (40.5%)	87 (57.2%)	< 0.001
SCAS, %	108 (70.6%)	120 (78.4%)	116 (75.8%)	130 (85.5%)	0.017
CRP, mg/L	0.99 (0.40–1.44)	1.58 (1.07–2.62)	2.48 (1.55–3.84)	4.51 (2.55–7.73)	< 0.001
NEUT, ×10^9/L	3.66 (2.99–4.54)	3.80 (3.04–5.00)	4.20 (3.34–5.46)	4.73 (3.80–6.46)	< 0.001
PLT, ×10^9/L	223.00 (191.00–262.00)	230.00 (193.00–277.00)	218.00 (184.00–253.00)	230.50 (189.75–267.25)	0.269
RBC, ×10^12/L	4.69 ± 0.50	4.75 ± 0.52	4.74 ± 0.52	4.82 ± 0.54	0.186
Hb, g/L	141.00 (130.00–155.00)	146.00 (134.00–154.00)	141.00 (131.00–153.00)	148.50 (136.75–158.00)	0.037
Hct, %	43.56 ± 4.70	44.24 ± 4.56	43.45 ± 4.59	44.21 ± 4.85	0.299
HbA1c, %	5.70 (5.50–6.00)	5.90 (5.60–6.40)	6.30 (5.80–7.20)	7.05 (5.90–8.90)	< 0.001
FPG, mmol/L	5.35 (5.01–5.74)	5.81 (5.30–6.50)	6.54 (5.56–8.02)	8.25 (6.27–11.35)	< 0.001
TC, mmol/L	4.64 (3.92–5.29)	4.80 (4.14–5.53)	5.04 (4.29–5.56)	5.08 (4.55–5.95)	< 0.001
TG, mmol/L	1.28 (0.98–1.55)	1.86 (1.43–2.35)	2.31 (1.83–2.98)	3.41 (2.39–4.67)	< 0.001
HDL-C, mmol/L	1.15 (1.02–1.30)	1.04 (0.90–1.20)	0.97 (0.86–1.08)	0.88 (0.76–1.01)	< 0.001
LDL-C, mmol/L	2.68 (2.12–3.13)	2.68 (2.14–3.41)	2.74 (2.08–3.28)	2.50 (1.92–3.16)	0.306
non-HDL-C, mmol/L	3.47 (2.88–4.06)	3.77 (3.16–4.42)	3.98 (3.27–4.59)	4.17 (3.66–5.06)	< 0.001
ALB, g/L	43.20 (41.20–45.40)	43.60 (41.20–45.40)	42.50 (39.60–44.70)	42.50 (39.08–44.75)	0.002
ALT, U/L	22.00 (15.00–33.00)	26.00 (20.00–38.00)	23.00 (16.00–34.00)	30.50 (20.00–45.00)	< 0.001
AST, U/L	21.00 (18.00–25.00)	22.00 (18.00–27.00)	21.00 (17.00–29.00)	24.50 (18.00–37.00)	< 0.001
BUN, mmol/L	5.34 (4.39–6.27)	5.36 (4.60–6.80)	5.54 (4.70–6.58)	5.56 (4.60–6.55)	0.342
UA, μmol/L	348.00 (301.00–402.10)	355.50 (290.10–418.90)	339.80 (296.20–414.30)	396.20 (332.30–461.58)	< 0.001
eGFR, mL/min/1.73 m²	102.01 (94.90–108.27)	101.08 (91.40–108.41)	99.15 (90.43–108.27)	100.07 (91.22–111.69)	0.490
Hcy, μmol/L	12.00 (10.00–15.00)	12.00 (10.00–17.00)	13.00 (10.00–17.00)	12.00 (10.00–15.00)	0.574
TSH, mIU/L	1.70 (1.19–2.45)	1.59 (1.12–2.55)	1.71 (1.09–2.50)	1.59 (1.20–2.35)	0.902
FIB, g/L	2.65 (2.26–3.13)	2.85 (2.39–3.33)	2.93 (2.56–3.57)	3.29 (2.56–3.96)	< 0.001
CTI	8.58 (8.37–8.81)	9.27 (9.14–9.40)	9.82 (9.68–9.98)	10.58 (10.34–10.88)	< 0.001
CHD [n(%)]	44 (28.8%)	90 (58.8%)	120 (78.4%)	133 (87.5%)	< 0.001

P < 0.05 was considered statistically significant.

Data are presented as mean (SD), median [IQR], or n (%). BMI, body mass index; SCAS, subclinical carotid atherosclerosis; CRP, C-reactive protein; NEUT, neutrophil count; PLT, platelet count; RBC, red blood cell count; Hb, hemoglobin; Hct, hematocrit; HbA1c, glycated hemoglobin; FPG, fasting plasma glucose; TC, total cholesterol; TG, triglycerides; HDL-C, high-density lipoprotein cholesterol; LDL-C, low-density lipoprotein cholesterol; non-HDL-C, non-high-density lipoprotein cholesterol; ALB, albumin; ALT, alanine aminotransferase; AST, aspartate aminotransferase; UA, uric acid; eGFR, estimated glomerular filtration rate; Hcy, homocysteine; TSH, thyroid-stimulating hormone; FIB, fibrinogen; CHD, coronary heart disease.

### 3.3 Association between CTI and CHD

As presented in **Table 3**, CTI was independently related to the odds of CHD in patients with MASLD. When CTI was analyzed as a continuous variable, a significant association was observed in the crude model (Model 1: OR 4.37, 95% CI 3.29–5.81, P < 0.001) and in the partially adjusted model (Model 2: OR 4.17, 95% CI 3.04–5.71, P < 0.001). This relationship remained significant after further adjustment in Model 3 (OR 2.48, 95% CI 1.69–3.63, P < 0.001).

**Table 3:** Association between CTI and CHD risk in patients with MASLD.

CTI	Model 1		Model 2		Model 3	
	OR (95% CI)	P value	OR (95% CI)	P value	OR (95% CI)	P value
Overall	4.37 (3.29-5.81)	<0.001	4.17 (3.04-5.71)	<0.001	2.48 (1.69-3.63)	<0.001
Q1, < 8.997	Ref. (1.0)		Ref. (1.0)		Ref. (1.0)	
Q2, 8.997-9.546	3.54 (2.20-5.69)	<0.001	3.15 (1.90-5.22)	<0.001	2.44 (1.41-4.24)	0.002
Q3, 9.546-10.158	9.01 (5.35-15.16)	<0.001	7.84 (4.47-13.75)	<0.001	4.63 (2.44-8.78)	<0.001
Q4, ≥ 10.158	17.34 (9.57-31.43)	<0.001	15.40 (8.00-29.64)	<0.001	4.58 (2.09-10.02)	<0.001
P for trend		<0.001		<0.001		<0.001

Model 1, unadjusted.

Model 2, adjusted for age, gender, BMI, smoking status, hypertension, and diabetes mellitus.

Model 3, adjusted for model 2 + NEUT, HbA1c, TC, HDL-C, ALB, ALT, AST, and UA.

OR, odds ratio; 95% CI, 95% confidence interval; BMI, body mass index; NEUT, neutrophil count; HbA1c, glycated hemoglobin; TC, total cholesterol; HDL-C, high-density lipoprotein cholesterol; ALB, albumin; ALT, alanine aminotransferase; AST, aspartate aminotransferase; UA, uric acid.

When CTI was further divided into quartiles, higher quartiles were associated with greater odds of CHD than the lowest quartile in all three models. In Model 3, the ORs for CHD were 2.44 (95% CI 1.41–4.24, P = 0.002) for Q2, 4.63 (95% CI 2.44–8.78, P < 0.001) for Q3, and 4.58 (95% CI 2.09–10.02, P < 0.001) for Q4, with a significant trend across quartiles (P for trend < 0.001).

**Figure 1:**
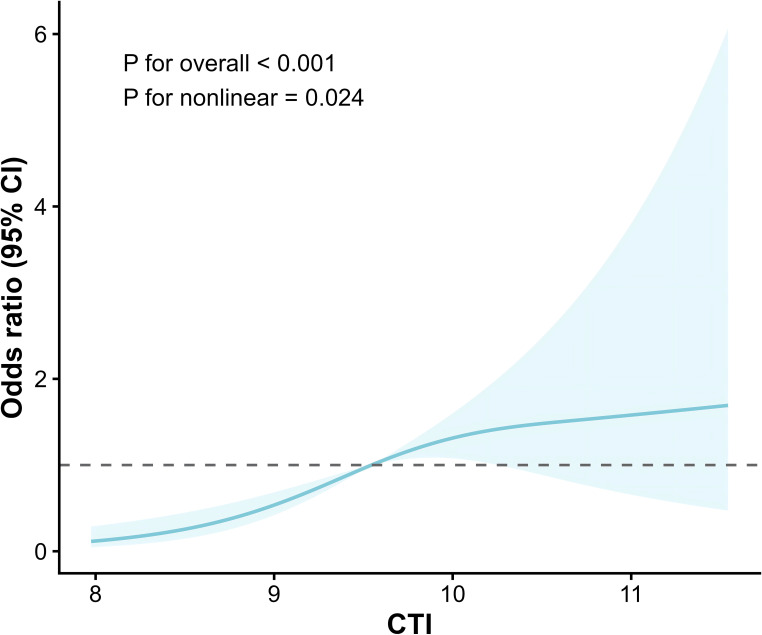
RCS curve for CTI in relation to CHD in patients with MASLD. Model adjusted for age, gender, body mass index, smoking status, hypertension, diabetes mellitus, neutrophil count, glycated hemoglobin, total cholesterol, high-density lipoprotein cholesterol, serum albumin, alanine aminotransferase, aspartate aminotransferase, and uric acid. OR, odds ratio; 95% CI, 95% confidence interval; CTI, C-reactive protein–triglyceride–glucose index; MASLD, metabolic dysfunction-associated steatotic liver disease; CHD, coronary heart disease.

Restricted cubic spline analysis adjusted for the same covariates as Model 3 showed a significant non-linear association between CTI and CHD in patients with MASLD (P for overall < 0.001; P for nonlinear = 0.024). The odds of CHD increased with increasing CTI, particularly within the middle-to-high CTI range, and then tended to level off at higher CTI values (**Figure 1**).

**Figure 2:**
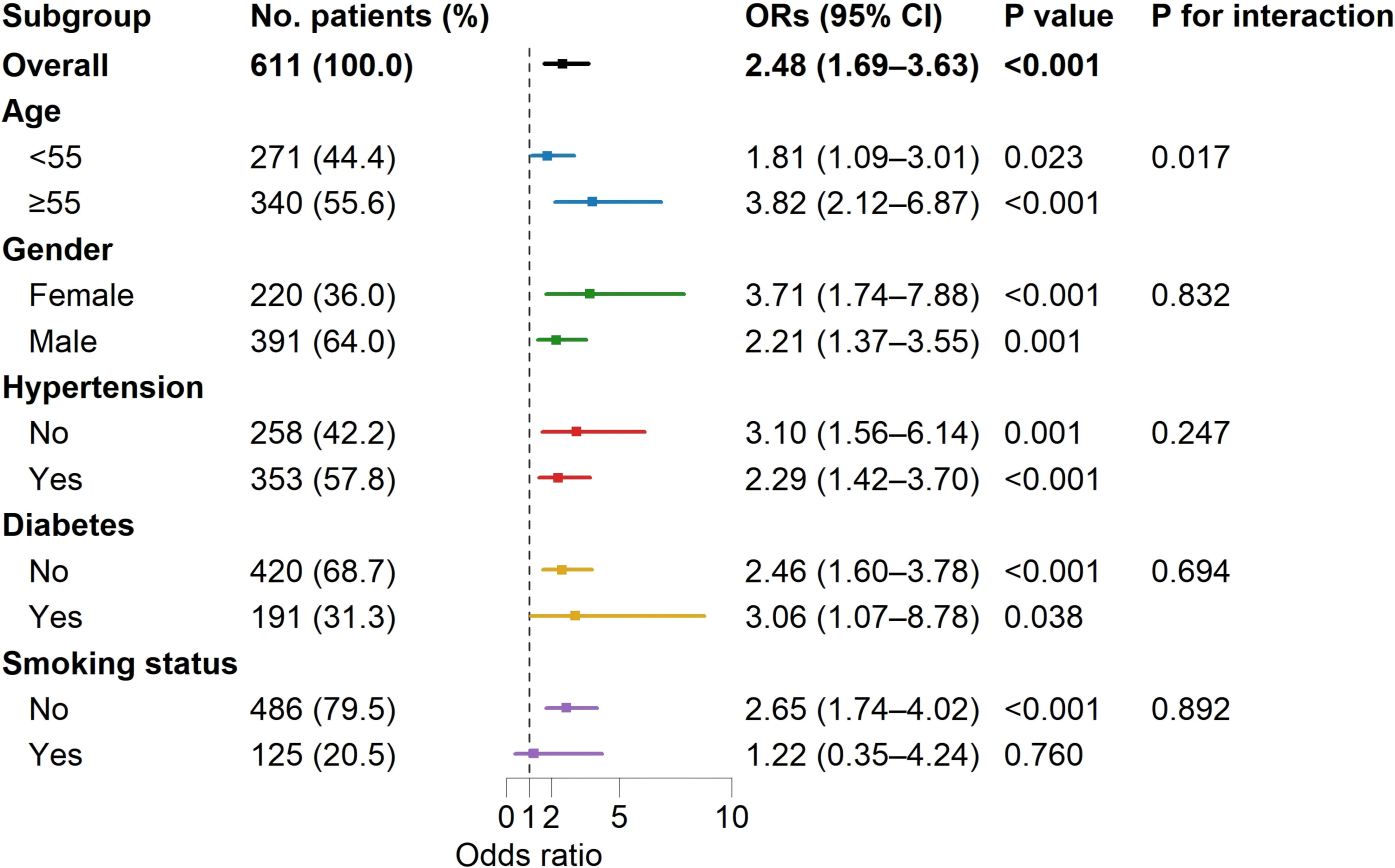
Associations between CTI and CHD in different subgroups. The forest plot illustrates the associations between CTI and CHD across various subgroups. Each subgroup analysis was adjusted for all factors except the stratification variable itself, including age, gender, body mass index, smoking status, hypertension, diabetes mellitus, neutrophil count, glycated hemoglobin, total cholesterol, high-density lipoprotein cholesterol, serum albumin, alanine aminotransferase, aspartate aminotransferase, and uric acid. OR, odds ratio; 95% CI, 95% confidence interval; CTI, C-reactive protein–triglyceride–glucose index; CHD, coronary heart disease.

Subgroup analyses were conducted using multivariable logistic regression stratified by age, sex, hypertension, diabetes mellitus, and smoking status (**Figure 2**). A positive association between CTI and CHD was observed across most subgroups, and no significant interaction was found for sex, hypertension, diabetes mellitus, or smoking status (all P for interaction > 0.05). However, a significant interaction was detected for age (P for interaction = 0.017). The association between CTI and CHD was more pronounced in participants aged ≥55 years (OR 3.82, 95% CI 2.12–6.87) than in those aged <55 years (OR 1.81, 95% CI 1.09–3.01).

### 3.4 Association of CTI with CHD severity

**Figure 3:**
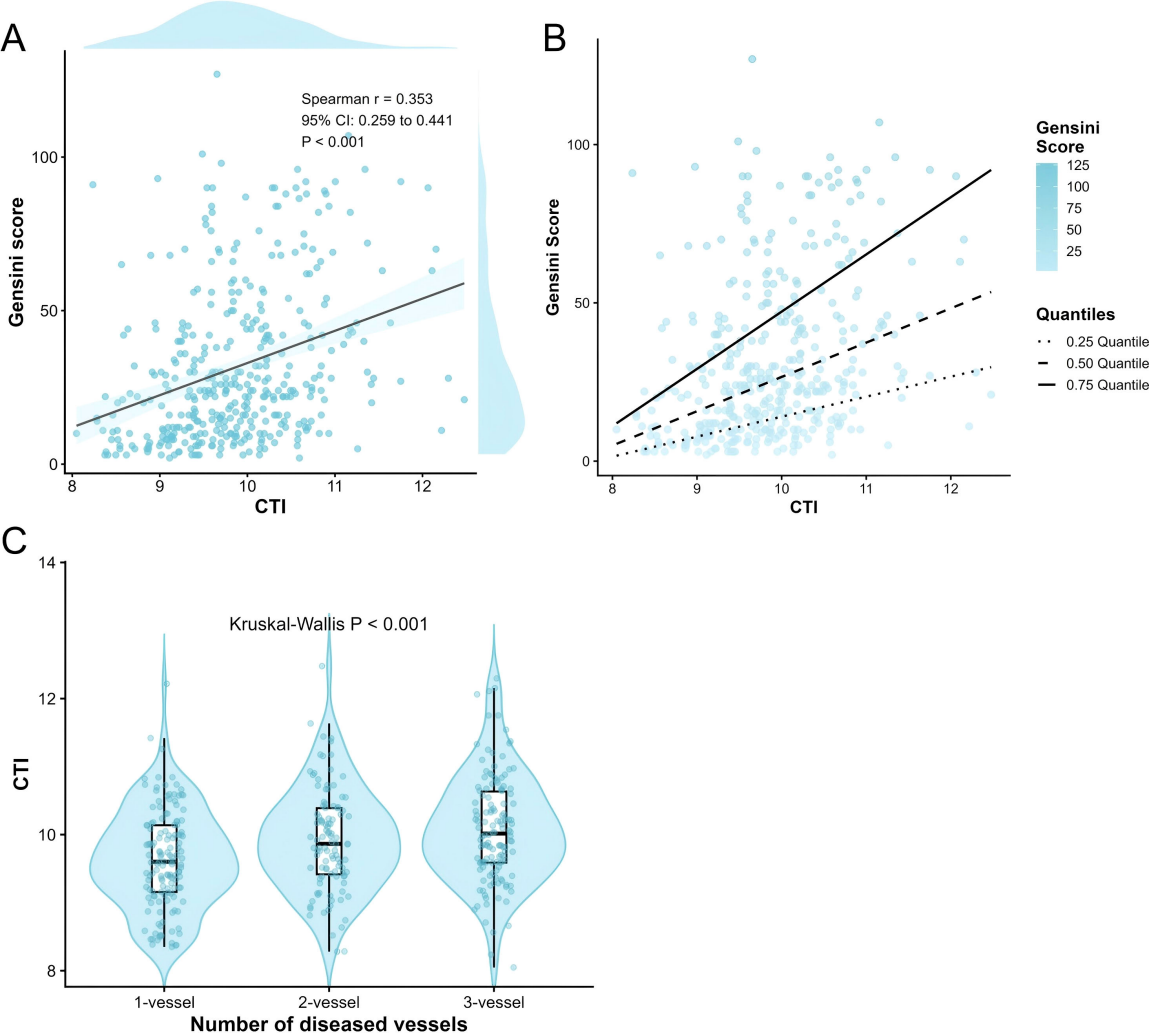
Association between CTI and coronary lesion severity in patients with MASLD and CHD. **(A)** Spearman correlation analysis between CTI and the Gensini score. The scatter plot shows a positive correlation between CTI and the Gensini score, with marginal density plots displaying the distributions of both variables. **(B)** Quantile regression analysis of the association between CTI and the Gensini score at the 0.25, 0.50, and 0.75 quantiles. **(C)** Distribution of CTI levels according to the number of diseased vessels. Differences among groups were assessed using the Kruskal–Wallis test. CTI, C-reactive protein–triglyceride–glucose index; MASLD, metabolic dysfunction-associated steatotic liver disease; CHD, coronary heart disease; 95% CI, 95% confidence interval.

Spearman’s rank correlation revealed a modest but statistically significant positive association between CTI and the Gensini score (r = 0.353, P < 0.001) (**Figure 3A**). Consistent with the correlation analysis, quantile regression further demonstrated that the positive association between CTI and Gensini score was consistent across different severity strata of CHD (**Figure 3B**). Specifically, the magnitude of the association was most pronounced at the 0.75th quantile, suggesting that the association between CTI and the Gensini score may be more pronounced among patients with more severe coronary stenosis. Furthermore, the Kruskal-Wallis test revealed that CTI levels increased progressively across subgroups classified by the number of diseased vessels [1-vessel: 9.60 (IQR 9.16–10.14), 2-vessel: 9.86 (IQR 9.42–10.39], 3-vessel: 10.02 [IQR 9.59–10.63); P < 0.001] (**Figure 3C**). Taken together, these observations imply that CTI may capture the joint burden of inflammatory and metabolic alterations related to atherosclerosis in patients with CHD.

### 3.5 Comparison of discriminative ability among CTI, TyG, TyG-BMI, METS-IR, and TG/HDL-C

**Figure 4:**
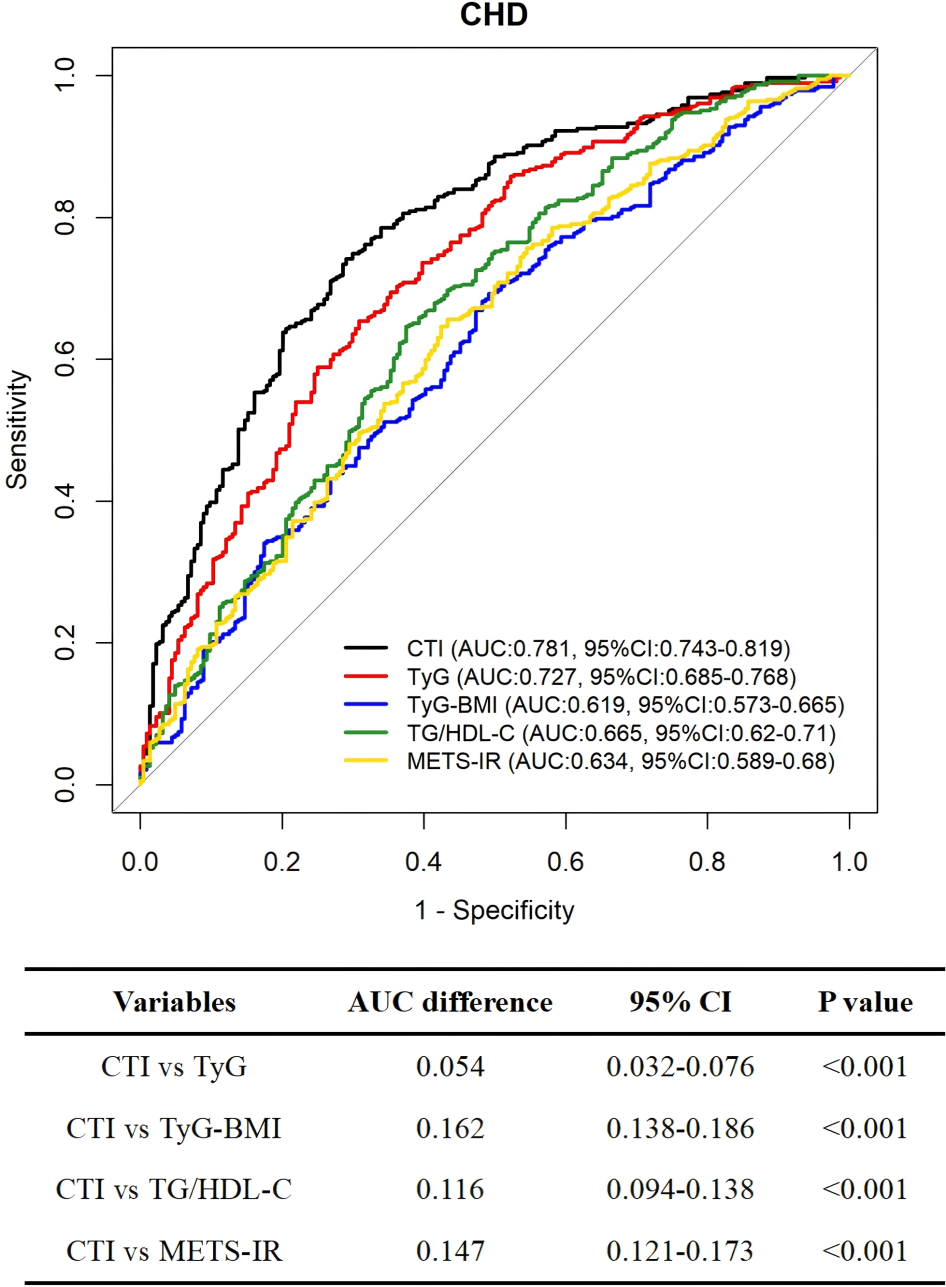
Comparison of discriminative ability between CTI, TyG, TyG-BMI, TG/HDL-C, and METS-IR using DeLong’s test. CTI, C-reactive protein–triglyceride–glucose index; TyG, triglyceride–glucose index; METS-IR, metabolic score for insulin resistance; TG, triglycerides; HDL-C, high-density lipoprotein cholesterol; TyG-BMI, triglyceride glucose-body mass index. The ROC curves of the five indices are shown, and the AUCs with 95% CIs are listed in the lower right corner.

ROC analysis indicated that CTI showed moderate discriminative performance for identifying CHD in patients with MASLD. The corresponding AUC was 0.781 (95% CI: 0.734–0.819), and the optimal cut-off point was 9.386, with sensitivity and specificity values of 74.2% and 71.0%, respectively. DeLong analysis further showed that CTI performed better than the TyG index (AUC difference: 0.054, 95% CI: 0.032–0.076, P < 0.001), TyG-BMI (AUC difference: 0.162, 95% CI: 0.138–0.186, P < 0.001), TG/HDL-C (AUC difference: 0.116, 95% CI: 0.094–0.138, P < 0.001), and METS-IR (AUC difference: 0.147, 95% CI: 0.121–0.173, P < 0.001) (**Figure 4**).

### 3.6 Development and validation of the nomogram

#### 3.6.1 Selection of candidate predictors

**Figure 5:**
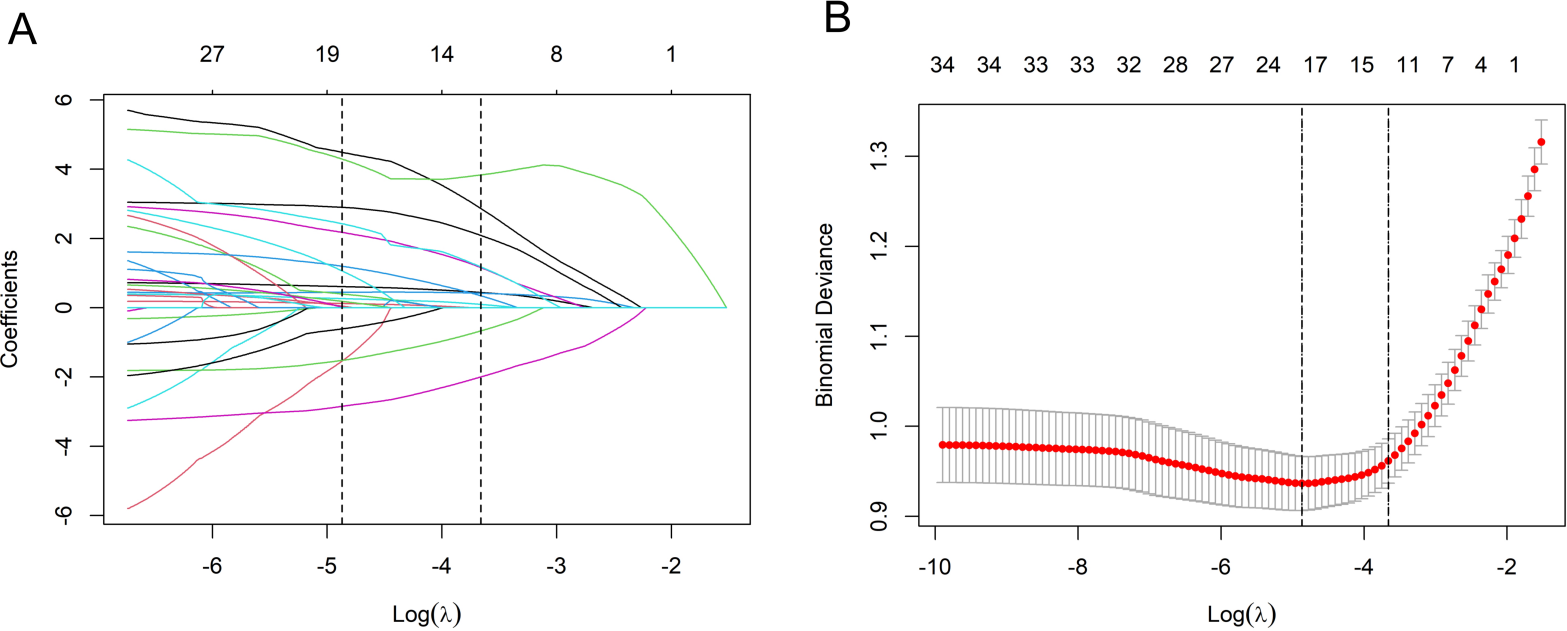
Variable filtering process of LASSO regression: **(A)** coefficient shrinkage path plot; **(B)** cross-validation plot.

LASSO regression with 10-fold cross-validation was first applied to screen candidate variables for CHD, with lambda.1se selected as the optimal tuning parameter (**Figure 5**). Eleven non-zero coefficient variables were identified: smoking status, SCAS, gender, age, NEUT, FPG, HDL-C, non-HDL-C, ALB, UA, and CTI. Because FPG contributes to CTI through the TyG component, it was excluded from the final model to reduce redundancy, avoid predictor overlap, and improve model stability.

Variables retained by the LASSO model were subsequently included in a multivariable logistic regression analysis. Smoking status (P = 0.020), age (P < 0.001), NEUT (P < 0.001), non-HDL-C (P = 0.005), ALB (P < 0.001), CTI (P < 0.001), and SCAS (P = 0.013) were finally identified as independent factors associated with CHD in patients with MASLD (**Table 4**).

**Table 4:** Multivariate logistic analyses of variables associated with CHD in MASLD.

Variable	β	SE	Wald Z	OR	95% CI	P value
Gender	0.517	0.267	1.935	1.676	0.993–2.829	0.053
Smoking status	0.714	0.307	2.326	2.041	1.119–3.725	0.020
Age	0.046	0.012	3.779	1.047	1.022–1.072	<0.001
NEUT	0.267	0.075	3.580	1.306	1.128–1.512	<0.001
HDL-C	-0.711	0.468	-1.520	0.491	0.196–1.229	0.129
non-HDL-C	0.310	0.110	2.831	1.364	1.1–1.691	0.005
ALB	-0.138	0.039	-3.586	0.871	0.808–0.939	<0.001
UA	0.001	0.001	1.119	1.001	0.999–1.004	0.263
CTI	1.163	0.178	6.543	3.198	2.258–4.53	<0.001
SCAS	0.627	0.253	2.483	1.872	1.141–3.072	0.013

S.E., standard error; Z, Z-score; OR, odds ratio; CI, confidence interval.

#### 3.6.2 Nomogram construction and validation

**Figure 6:**
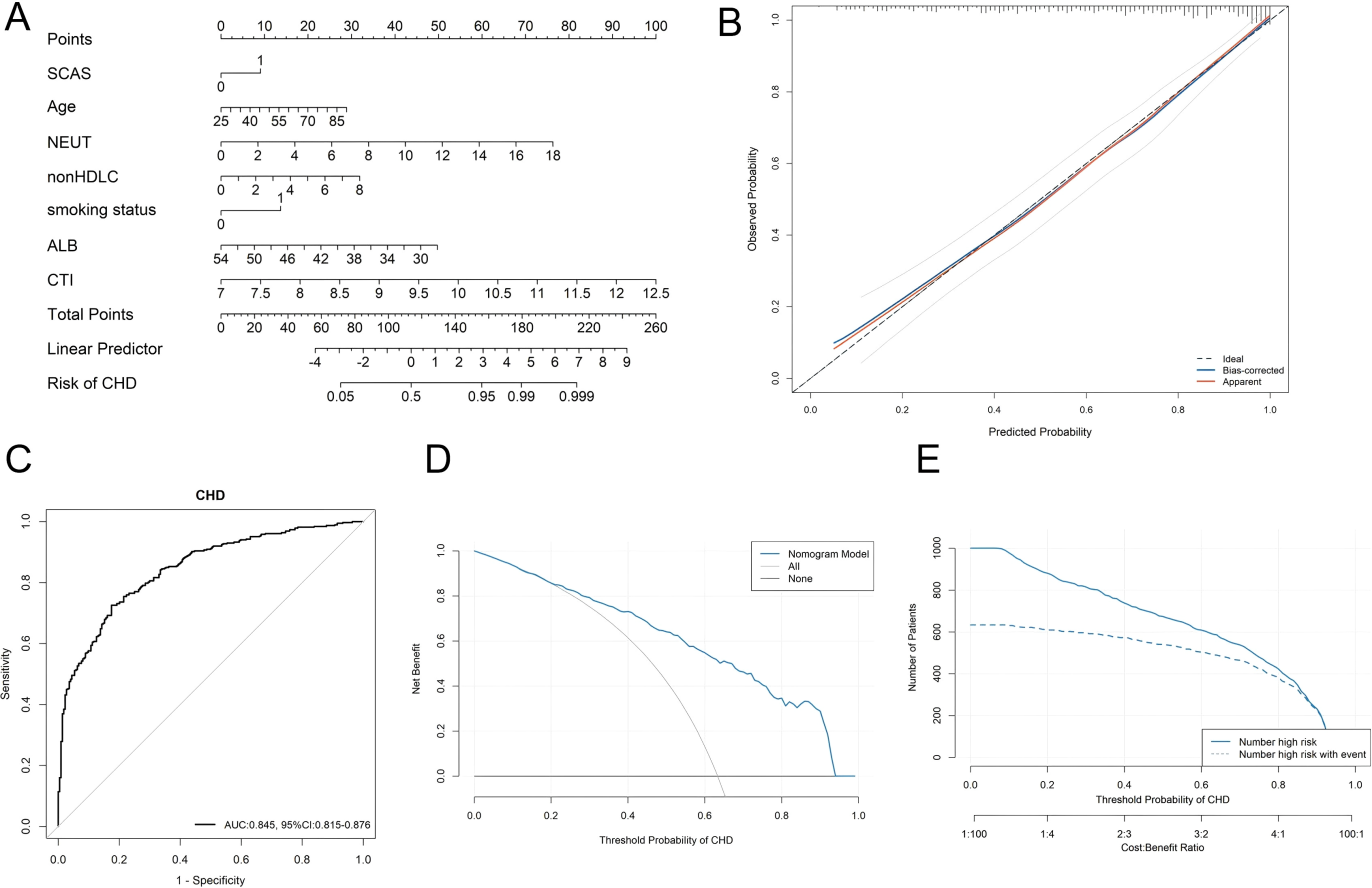
**(A)** Nomogram for assessing CHD in patients with MASLD. Nomogram incorporating SCAS, age, NEUT, non-HDL-C, smoking status, ALB, and CTI to estimate the probability of CHD in patients with MASLD. For each patient, the score for each predictor is assigned according to the points scale. The total points are then projected onto the bottom scale to estimate the individual probability of CHD. **(B)** Calibration curve of the nomogram. The calibration curve assessed the agreement between nomogram-predicted probabilities and observed CHD outcomes. The x-axis represents the predicted probability of CHD, and the y-axis represents the observed probability. The apparent and bias-corrected curves indicate the calibration performance of the model. **(C)** Receiver operating characteristic (ROC) curve of the nomogram. The area under the curve was 0.845, with a 95% confidence interval of 0.815-0.876. **(D)** Decision curve analysis (DCA) of the nomogram. The y-axis represents the net benefit. The horizontal solid line represents the treat-none strategy, and the diagonal solid line represents the treat-all strategy. The nomogram curve indicates its clinical utility across different threshold probabilities. **(E)** Clinical impact curve (CIC) of the nomogram. The y-axis represents the number of patients. The solid line indicates the total number of high-risk patients for CHD, and the dashed line indicates true CHD events within the high-risk group across different threshold probabilities and cost-benefit ratios. CHD, coronary heart disease; MASLD, metabolic dysfunction-associated steatotic liver disease; SCAS, subclinical carotid atherosclerosis; NEUT, neutrophil count; non-HDL-C, non-high-density lipoprotein cholesterol; ALB, albumin; CTI, C-reactive protein–triglyceride–glucose index.

A nomogram model integrating SCAS, age, NEUT, non-HDL-C, smoking status, ALB, and CTI was established for individualized CHD assessment in MASLD patients (**Figure 6A**). As shown in **Figure 6C**, ROC curve analysis demonstrated good discriminative ability of the nomogram, with an AUC of 0.845 (95% CI: 0.815–0.876). Bootstrap internal validation with 1,000 resamples showed that the apparent and bias-corrected calibration curves of the nomogram were both close to the ideal reference line, indicating good agreement between predicted and observed CHD probabilities (**Figure 6B**). DCA indicated that the nomogram yielded a favorable net benefit over a clinically relevant range of threshold probabilities (**Figure 6D**). CIC analysis further showed that the model was able to identify most patients classified as high risk across a broad range of thresholds, with a favorable balance between true-positive and false-positive predictions (**Figure 6E**). The incremental value of CTI was further evaluated by comparing the final nomogram model without CTI (SCAS, age, NEUT, non-HDL-C, smoking status, and ALB) with the full model including CTI. Adding CTI increased the AUC from 0.785 to 0.845, with a ΔAUC of 0.061 (95% CI 0.037–0.084; DeLong P < 0.001). CTI also improved risk reclassification, with an IDI of 0.105 (95% CI 0.066–0.149) and a continuous NRI of 0.639 (95% CI 0.491–0.851).

## 4 Discussion

Defined by excess lipid accumulation within hepatocytes, MASLD ranks among the most prevalent chronic liver disorders globally (35). While its core feature is hepatic steatosis, it is now widely viewed as a marker of systemic cardiometabolic dysfunction. Research increasingly shows that MASLD is not confined to the liver but correlates with numerous extrahepatic complications and elevated mortality risk (10). In particular, MASLD has been identified as a significant risk factor for both fatal and non-fatal cardiovascular diseases (6). Cardiovascular complications, including myocardial infarction, atrial fibrillation, and heart failure, may become clinically evident even before overt hepatic events, highlighting the often underrecognized cardiovascular burden associated with MASLD (36). Consequently, prompt assessment of cardiovascular risk and timely intervention in these patients are essential to enhance life quality and reduce mortality risk.

CTI combines CRP with the TyG index and therefore integrates inflammatory, glycemic, and triglyceride-related metabolic information. In the present study, higher CTI levels were associated with increased odds of CHD in patients with MASLD after adjustment for classic cardiovascular risk factors, including age, gender, smoking status, HT, and DM. Higher CTI levels were also associated with greater coronary artery disease severity, as reflected by the Gensini score and diseased-vessel number.

This index was first introduced by Ruan et al. for prognostic assessment in patients with cancer (37). Evidence from large population-based studies has consistently supported the cardiovascular relevance of CTI. In a nationwide prospective cohort of 17,705 participants with cardiovascular–kidney–metabolic (CKM) syndrome stages 0–3, Ou et al. reported that elevated CTI was linked to a greater risk of incident CVD and all-cause mortality (38). Consistent with these findings, Ma et al. followed 4,157 middle-aged and elderly Chinese participants and observed that a higher long-term CTI burden, as well as persistently high CTI trajectories, was associated with a higher incidence of new-onset CVD (39). In another nationwide cohort including 5,111 individuals with CKM syndrome, Chen et al. showed that both baseline and cumulative CTI were related to subsequent CVD risk, and that cumulative CTI provided better predictive performance than cumulative TyG (40). In addition, Hou et al. found that CTI was useful in predicting three-vessel coronary artery disease, indicating that this index may help identify patients with more advanced coronary lesions (41). Beyond cardiovascular outcomes, Zhou et al. reported, in 3,488 American adults, that elevated CTI was linked to NAFLD and liver fibrosis and showed better diagnostic accuracy than either TyG or hs-CRP used alone (42). Taken together, these studies indicate that CTI may serve as a practical indicator linking inflammation, insulin resistance, and adverse cardiometabolic outcomes. However, most of the available evidence has been derived from the general population, with limited data available for specific groups like individuals with MASLD.

MASLD and CHD are closely interconnected through shared pathophysiological mechanisms, among which IR and chronic low-grade inflammation appear to be particularly important (10). The association between CTI and CHD in MASLD may be biologically plausible because the two components of CTI map onto key pathways linking hepatic metabolic dysfunction to coronary atherosclerosis. The TyG component of CTI links this index to insulin resistance, a central driver of hepatic lipid accumulation in MASLD. When insulin signaling is impaired, the suppression of adipose lipolysis becomes weaker, resulting in increased delivery of free fatty acids to the liver (43). In parallel, reduced fatty acid oxidation and insufficient VLDL-mediated lipid disposal relative to lipid influx lead to hepatic triglyceride accumulation and lipotoxic stress (44). Importantly, this lipid overload may have direct biological consequences (45). Toxic lipid species trigger inflammatory pathways such as NF−κB and the NLRP3 inflammasome, leading to increased cytokine production and recruitment of immune cells into the liver (46, 47). Consequently, hepatic insulin resistance worsens through NF−κB activation and ER stress, reinforcing a harmful feedback loop between IR and inflammation (48). Meanwhile, IR reduces endothelial nitric oxide bioavailability, impairs endothelial function, promotes vascular stiffness, and shifts cardiomyocyte metabolism toward greater fatty acid dependence, which may increase myocardial vulnerability to ischemia (49). Beyond its role in the TyG index, TG may also capture a broader proatherogenic lipoprotein phenotype rather than isolated hypertriglyceridemia. Amigó et al. recently showed that increasing TG concentrations were associated with global lipoprotein derangement and a more proatherogenic plasma lipoprotein profile, including increased triglyceride-rich lipoproteins, smaller LDL particles, higher remnant cholesterol, and altered lipid composition of LDL and HDL particles (50). These changes may promote cholesterol deposition, vascular injury, vascular inflammation, and plaque progression. This mechanism is particularly relevant in MASLD, where hepatic lipid overload and insulin resistance may increase VLDL secretion and worsen systemic lipoprotein remodeling.

CRP, the other component of CTI, sensitively captures systemic inflammatory activity and may also be related to atherosclerotic plaque development and rupture. It has been shown to be strongly associated with elevated cardiovascular risk (51, 52). As a metabolic disorder, MASLD interferes with immune regulation and sustains chronic low−grade inflammation, thereby promoting the development of CHD (53). MASLD-related gut dysbiosis and pro-inflammatory dietary exposures may increase circulating lipopolysaccharide and gut-derived metabolites such as trimethylamine N-oxide, thereby contributing to systemic inflammation, endothelial dysfunction, and atherosclerotic progression (54). Notably, recent experimental evidence also suggests that steatotic hepatocyte−derived small extracellular vesicles may accelerate atherogenesis through the miR−30a−3p/ABCA1 axis (55). Chronic inflammation promotes LDL oxidation, monocyte recruitment, macrophage activation, and foam cell formation, thereby facilitating the initiation and progression of coronary atherosclerotic plaques (56, 57). Persistent inflammation may also contribute to microcalcification and plaque vulnerability through repeated inflammatory injury and matrix degradation mediated by metalloproteinases, ultimately increasing the likelihood of plaque instability and coronary events (58). These mechanistic links are consistent with recent evidence emphasizing the role of inflammatory profiling in identifying subclinical atherosclerotic burden and improving cardiovascular risk stratification (59).

Co-exposure effects and mutual mediation between CRP and the TyG index have been reported in relation to CVD (cardiovascular disease) events (60). In addition, Ma et al. observed a significant interaction between the TyG index and CRP in predicting major adverse cardiovascular events (MACE) risk among patients with acute coronary syndrome undergoing percutaneous coronary intervention (61). Given that cardiovascular risk in MASLD is not driven by IR alone, but also by systemic inflammation and endothelial injury, CTI may provide a more integrated approach for CHD risk stratification in this population. This may partly explain our ROC findings, in which CTI showed better discriminative performance than TyG, TyG-BMI, TG/HDL-C, and METS-IR for identifying CHD in patients with MASLD.

The RCS analysis further suggested that the association between CTI and CHD was non-linear rather than strictly linear. The odds of CHD increased more noticeably within the middle-to-high CTI range and then tended to plateau at higher levels. This pattern may indicate that once inflammatory and metabolic disturbances reach a certain threshold, the incremental discriminative value of further CTI elevation becomes less pronounced. Subgroup analyses showed generally consistent positive associations across gender, HT, DM, and smoking status, whereas age showed a significant modifying effect. The association was stronger in patients aged ≥55 years, possibly because older MASLD patients may have longer exposure to metabolic abnormalities and greater cumulative vascular injury. A similar age-related pattern has also been reported in metabolically heterogeneous populations (62). In addition, aging is often accompanied by more complex metabolic changes, and hormonal changes with aging, especially postmenopausal estrogen decline, may further aggravate metabolic dysfunction and vascular injury (63–65). This finding highlights that age should be considered when evaluating metabolic and cardiovascular risk in patients with MASLD.

Neutrophils, SCAS, age, smoking status, and non-HDL-C were also identified as factors independently associated with CHD, whereas ALB was independently and inversely associated with CHD. Among these variables, age, smoking status, and non-HDL-C are traditional CHD risk factors. In contrast, SCAS may reflect subclinical structural arterial injury and systemic atherosclerotic burden, providing imaging evidence of cumulative vascular damage that parallels coronary atherosclerosis (66, 67). Previous studies have suggested that incorporating carotid ultrasound markers of subclinical atherosclerosis into prediction models may improve the identification and risk stratification of CHD beyond conventional clinical risk factors. Thus, beyond conventional risk factors, subclinical vascular remodeling may also contribute to CHD susceptibility in patients with MASLD (68). Emerging evidence suggests that neutrophils may contribute to CHD beyond serving as markers of systemic inflammation. They may also participate in endothelial injury, foam-cell formation, plaque destabilization, NET-mediated immunothrombosis, and myocardial ischemia–reperfusion injury (69, 70). In addition, ALB plays an important role in maintaining plasma osmotic pressure and has anti-inflammatory and antioxidant properties (71). Lower ALB levels may reflect weakened inflammatory and oxidative defense, which could partly explain their inverse association with CHD (72).

Clinically, CTI may be a practical and accessible marker for CHD risk assessment in patients with MASLD. It is derived from routine laboratory parameters, is inexpensive, and can be easily calculated in daily practice. This may help prioritize MASLD patients who warrant closer cardiovascular evaluation, particularly in settings where coronary angiography is not suitable as a first-line assessment. The nomogram developed in this study showed favorable internal performance. Incremental value analyses showed that adding CTI to the model without CTI improved discrimination and risk reclassification, supporting its added value in CHD assessment among patients with MASLD.

Several limitations need to be considered. First, the retrospective cross-sectional design prevents causal inference. Second, this single-center study included patients who underwent coronary angiography according to clinical indications rather than an unselected MASLD population. Therefore, selection bias may exist, and the observed CHD prevalence and associations may not be directly generalizable to broader MASLD populations or other healthcare settings. Third, CTI was calculated using a single baseline measurement. Its components, including CRP, FPG, and TG, may fluctuate with acute stress, dietary intake, medication use, and biological rhythms. Thus, a single time-point value may not fully reflect the long-term inflammatory and metabolic status. Longitudinal studies should evaluate whether CTI trajectories are associated with CHD and coronary lesion severity in patients with MASLD. Fourth, MASLD was diagnosed mainly on the basis of ultrasonography rather than histology or advanced imaging, and detailed staging of liver disease was unavailable. Fifth, residual confounding cannot be completely excluded because physical activity, dietary patterns, genetic predisposition, socioeconomic status, and medication exposure were not adequately captured in this retrospective dataset. Finally, although the nomogram showed good performance in internal validation, external validation was not performed. This represents a major limitation and restricts the generalizability and immediate clinical applicability of the model. Before broader clinical use can be recommended, the nomogram should be externally validated in independent MASLD cohorts, preferably using diverse, prospective, multicenter designs to evaluate its robustness across different populations and healthcare settings. In addition, more detailed liver assessment and follow-up data on cardiovascular outcomes should be incorporated to better define the clinical value of CTI.

## 5 Conclusion

In conclusion, higher CTI was associated with CHD presence and greater coronary lesion burden in patients with MASLD. CTI may reflect combined inflammatory and metabolic disturbances related to coronary atherosclerosis and may support CHD risk assessment in this population. The nomogram showed favorable internal performance, but external validation in independent prospective multicenter MASLD cohorts is required before broader clinical application.

## Data Availability

The data analyzed in this study is subject to the following licenses/restrictions: The datasets analyzed during the current study are not publicly available due to privacy and ethical restrictions, but are available from the corresponding author on reasonable request. Requests to access these datasets should be directed to wuhuili660912@zzu.edu.cn.
